# An unhealing wound and subcutaneous nodules due to *Sporothrix globosa* after a cat bite

**DOI:** 10.1371/journal.pntd.0008859

**Published:** 2020-12-03

**Authors:** Yanbin Liu, Lina Liu, Mei Kang, Zhiyong Zong

**Affiliations:** 1 Center of Infectious Diseases, West China Hospital, Sichuan University, Chengdu, China; 2 Department of Laboratory Medicine, West China Hospital, Sichuan University, Chengdu, China; University of Iowa, UNITED STATES

## Abstract

A 51-year-old man with 3-month unhealing cat bite wound was diagnosed with sporotrichosis, a subacute-to-chronic infection caused by the worldwide endemic, dimorphic fungus *Sporothrix globosa*. The case would help clinicians to raise awareness of human sporotrichosis due to cat bites, which remains rare and is likely to be underrecognized and misdiagnosed.

## Case presentation

A 51-year-old man was admitted due to a wound at the right hand and progressive subcutaneous nodules at the right arm for 1 month. Three months prior to the admission, he was bitten at the right hand by a stray kitten. The cat was sick and died 6 days after the bite due to unknown diseases. The patient felt pain after the bite and immediately flushed the wound with tap water for several minutes but did not take other measures for dealing with the wound. He had no fever, and the wound healed 2 weeks after the bite. However, 1 month prior to the admission, the healed wound cracked with pus, and he found multiple subcutaneous nodules at the right arm. He felt nauseous occasionally but did not have fever, pain, vomiting, cough, dyspnea, pain, and headache. He went to local clinics and received antibiotics, but he did not know which agents were given. His wound appeared to be gradually healed again but became a verruca-like nodule, and new subcutaneous nodules emerged at the right arm. He went to the West China Hospital for further treatment. He had been a cottonsmith for many years and lived in Liangshan Region, Sichuan Province, Southwest China, with a subtropical climate. He had no travel history within the past few years. He was previously healthy with no underlying diseases such as diabetes. He drank alcohol occasionally and did not smoke cigarettes or use illicit substances.

**Fig 1:**
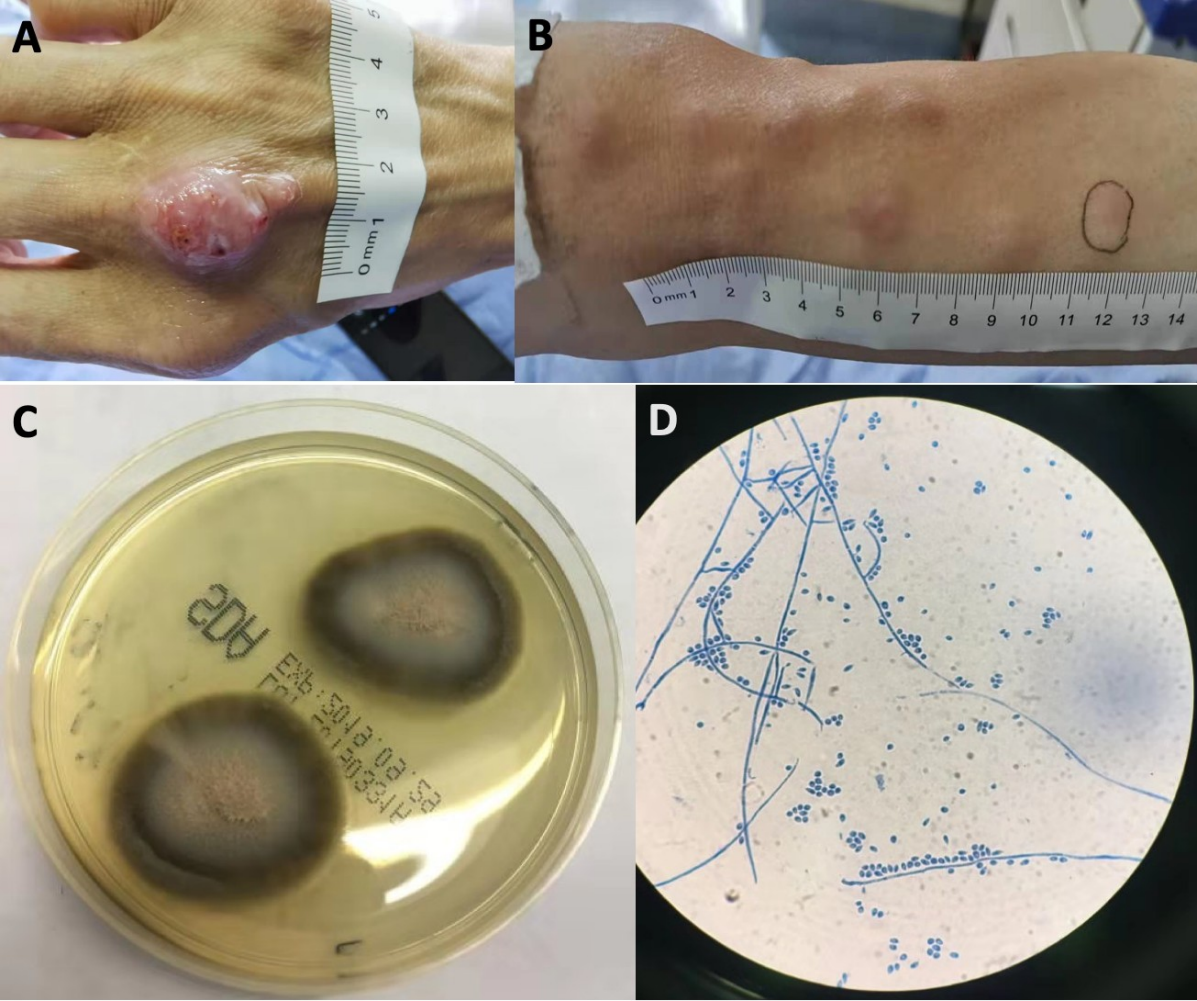
The wound and nodules 3 months after the cat bite and *Sporothrix globosa* recovered from the wound pus. (A) The wound of the right hand at 3 months. (B) Nodules at the right arm. (C) A macroscopic view of *S*. *globosa* is shown. This *S*. *globosa* strain forms dark colonies, which may be due to the ability to produce melanin in the culture media. (D) A microscopic view (lactophenol cotton blue stain, ×400 magnification) of *S*. *globosa* from Sabouraud Dextrose Agar.

On admission, he was afebrile. A 2 cm × 1.5 cm nodule-like wound was present at the back of the right hand (panel A, Fig 1) with mild tenderness, and pus emerged when the wound is pressed. About 20 subcutaneous nodules without erythema (about 1 to 1.5 cm in size; panel B, Fig 1) were detected at the right arm. There were no other remarkable findings on physical examination. The blood test on admission revealed white blood cells 5.24 × 10^9^/L (reference range 3.5 to 9.5 × 10^9^/L) with 55.5% neutrophils (reference range 40% to 75%) and 35.1% lymphocytes (reference range 20% to 50%), hemoglobin 159 g/L (reference range 115 to 150 g/L), and platelets 205 × 10^9^/L (reference range (100 to 300 × 10^9^/L). Serum biochemistry tests on admission showed that all values were in the normal range including total bilirubin 19.6 μmol/L (reference range 5 to 28 μmol/L), alanine aminotransferase 28 IU/L (reference range <50 IU/L), aspartate aminotransferase 24 IU/L (reference range <40 IU/L), albumin 43.7 g/L (reference range 40 to 55 g/L), globulin 27.2 g/L (reference range 20 to 40 g/L), urea 7.7 mmol/L (reference range 3.38 to 8.57 mmol/L), and creatine 57 μmol/L (reference range 53 to 140 μmol/L). He was married and denied any sexual activities outside the marriage. Serum tests for HIV, hepatitis B virus (HBV), hepatitis C virus (HCV), and syphilis were negative. An interferon gamma release assay for tuberculosis (Wantai BioPharm, Beijing, China) was negative. Pus was collected by pressing the wound after proper disinfection and was sent for culture.

Culture of the pus on Sabouraud Dextrose Agar (Autobio, Zhengzhou, Henan, China) at 28°C after 3 days grew *Sporothrix* spp. (Fig 1; a macroscopic view is shown in panel C, and a microscopic view [lactophenol cotton blue stain] is shown in panel D), which was also able to grow on self-prepared Potato Dextrose Agar at 30°C. The diagnosis of sporotrichosis was therefore established. Genome DNA of the strain was prepared using the sodium dodecyl sulfate (SDS) lysis method for yeast as described previously [1]. Partial sequences of the chitin synthase 1 gene *chs* and the DNA topoisomerase II gene *top2* were amplified from the strain as described previously [2,3], and amplicons were sequenced using the Sanger method. The partial sequence of *chs* (389 bp) and *top2* (632 bp) was 100% and 99.5% (629/632 bp), identical to the corresponding sequences of *Sporothrix globosa* CBS 120340 (GenBank accession no. LVYW01 [https://www.ncbi.nlm.nih.gov/assembly/GCA_001630435.1/]), respectively, but was 98.7% (381/386 bp) and 84.2% (532/632 bp) to *Sporothrix schenckii* 1099–18 (GenBank accession nos. XM_016735087.1 [https://www.ncbi.nlm.nih.gov/nuccore/XM_016735087.1/] and XM_016734889.1 [https://www.ncbi.nlm.nih.gov/nuccore/ XM_016734889.1/]). To further confirm the species identification, we designed 3 pairs of primers to amplify genes encoding DNA polymerase α subunit B (Sk/g-F1, 5′-CCATTGTTCCGTATGCCACGT; Sk/g-R1, 5′-CGGGCCCGAGGCGAACAT), DNA-directed RNA polymerase I subunit (Sk/g-F2, 5′-GTTCATCGTGGTAGTTTCT; Sk/g-R2, 5′-TCGAGCGCCGACTTGTTGATAAT), and DNA-directed RNA polymerase II subunit (Sk/g-F3, 5′-ATCCAGTCGGAGGTGTCGCTGGTGCGC; Sk/g-R3, 5′-CGCACCTTGACGTACCGCAG), and amplicons were sequenced. The obtained 572-bp, 830-bp, and 980-bp sequence was all 100% identical to that of *S*. *globosa* CBS 120340 (GenBank accession no. LVYW01) and was 94.4% (540/572 bp), 96.9% (804/830 bp), and 97.2% (953/980-bp) identical to that of *S*. *schenckii* 1099–18 (GenBank accession nos. XM_016732750.1 [https://www.ncbi.nlm.nih.gov/nuccore/XM_016732750.1/], XM_016729801.1 [https://www.ncbi.nlm.nih.gov/nuccore/XM_016729801.1/], and XM_016737181.1 [https://www.ncbi.nlm.nih.gov/nuccore/XM_016737181.1/]), respectively. The corresponding sequences of the strain in this study have been deposited in GenBank under accession nos. MN956987 and MT126697-MT126700. Therefore, it was confirmed that the strain belonged to *S*. *globosa*, which was previously known as clade III of *S*. *schenckii* [4] and is the prevalent *Sporothrix* species in Asia including China [5].

## Case discussion

This patient was bitten by a cat and had an unhealing wound at the bite, which also progressed into multiple ascending subcutaneous nodules. Infections associated with cat bites were the most likely diagnosis. The etiology of cat bite–associated infections is complicated and is often due to a mixture of aerobic and anaerobic microorganisms, which are commonly part of the cat oral microbiota and may also originate from the skin flora of the victim or the environment [6]. The most common microorganism recovered from infected wounds due to cat bites is *Pasteurella multocida*, while *Streptococcus* spp., *Staphylococcus* spp., *Fusobacterium* spp., *Porphyromonas* spp., and *Bacteroides* spp. are also relatively common [6,7]. However, infections due to these bacteria such as *P*. *multocida* usually progress rapidly and may lead to erythema and severe pain within hours after the bite [8]. This is not consistent with subacute onset of the disease and the initial healing without treatment after the cat bite in the patient. In addition, the cat was sick and died soon after the bite. Therefore, other pathogens should be suspected.

Sporotrichosis is a subacute-to-chronic infection caused by the worldwide endemic, dimorphic fungus *S*. *schenckii* [9], which was a complex comprising several closely related species [4]. Cat infected with the *S*. *schenckii* complex has been known as an important zoonotic source of human sporotrichosis since the 1980s [10]. After cat bite, the *S*. *schenckii* complex can inoculate in the wound and then cause ulcerated, verrucous, and nodular skin infection [6,9]. Human sporotrichosis due to cat bites has been reported in a few countries (Argentine, Australia, Brazil, Germany, India, Japan, Malaysia, Mexico, Panama, Spain, and the United States of America), but most cases were seen in Brazil [10,11], where sporotrichosis was mainly caused by *Sporothrix brasiliensis*, known as clade I of *S*. *schenckii* [4]. Outside Brazil, human sporotrichosis due to cat bites is rare and is likely to be underrecognized and misdiagnosed. Although *S*. *globosa* is the prevalent *Sporothrix* species, nearly all cat-associated cases were caused by *S*. *schenckii* in Asia [5]. However, this case suggests that *S*. *globosa* can also cause cat bite wound infection, and *S*. *schenckii* recovered from clinical samples may need to be subjected to precise species identification.

The patient received itraconazole 200 mg twice daily and terbinafine 250 mg once daily for 1 month [9]. The wound has completely healed, and the nodules at the right arm have largely resolved. Then, the patient received itraconazole 200 mg twice daily [9] alone for 6 months and remains well in the 6-month follow-up.

## Ethics statement

The patient in this manuscript provided written informed consent (as outlined in the PLOS consent form) to the publication of the case details.

**Key learning points**

- Unhealing cat bite wounds can be caused by infection of *Sporothrix*, a worldwide endemic, dimorphic fungus but are likely to be underrecognized and misdiagnosed.
- The *Sporothrix* species causing cat bite wound infection is usually reported as *Sporothrix schenckii*, but *S*. *schenckii* is actually a complex comprising several closely related species including *Sporothrix globosa*.
- *S*. *globosa* is prevalent in Asia and can cause cat bite wound infection.
- Cat bite wound infection due to *S*. *globosa* can be treated by itraconazole 200 mg twice daily.
